# Interaction of daytime and nighttime light exposure on objective sleep quality in patients with bipolar disorder: a cross-sectional analysis of the APPLE cohort

**DOI:** 10.1038/s41398-025-03549-3

**Published:** 2025-08-18

**Authors:** Yuichi Esaki, Kenji Obayashi, Keigo Saeki, Kiyoshi Fujita, Nakao Iwata, Jamie M. Zeitzer, Tsuyoshi Kitajima

**Affiliations:** 1 Department of Psychiatry, Okehazama Hospital, Toyoake, Aichi Japan; 2 Department of Psychiatry, Fujita Health University School of Medicine, Toyoake, Aichi Japan; 3 Department of Epidemiology, Nara Medical University School of Medicine, Nara, Japan; 4 The Neuroscience Research Center, Toyoake, Aichi Japan; 5 Department of Psychiatry and Behavioral Sciences, Stanford University, Stanford, CA USA; 6 Mental Illness Research Education and Clinical Center, VA Palo Alto Health Care System, Palo Alto, CA USA

**Keywords:** Bipolar disorder, Human behaviour

## Abstract

Light plays a crucial role in regulating nocturnal sleep patterns. This cross-sectional study evaluated the potential association between levels of light exposure in real-life settings and sleep parameters in individuals with bipolar disorder. We included 204 ambulatory individuals with bipolar disorder who participated in the APPLE (Association between Pathology of Bipolar Disorder and Light Exposure in Daily Life) cohort study. Daytime illuminance and sleep were assessed using actigraphy over a seven-day period. In addition, a portable light meter was used to evaluate the illuminance levels in the bedroom during nighttime. The median values of daytime illuminance and nighttime illuminance were 221.8 lux (interquartile range: 150.9–306.9 lux) and 2.3 lux (0.3–9.6 lux), respectively. Multivariable linear regression analyses, adjusting for potential confounders, revealed a significant association between greater daytime illuminance and higher sleep efficiency as well as shorter sleep onset latency and wake after sleep onset. Moreover, the interaction term of daytime and nighttime illuminance demonstrated a significant correlation with sleep efficiency (95% confidence interval [CI], −10.45 to −2.17; *P* = 0.003), sleep onset latency (95% CI, 0.18 to 0.91; *P* = 0.004), and wake after sleep onset (95% CI, 13.47 to 50.1; *P* < 0.001). Our findings indicate the existence of a significant positive correlation between daytime light exposure and sleep parameters in individuals with bipolar disorder. The interaction of increased daytime light and decreased nighttime light appears to be positively associated with sleep quality.

## Introduction

Bipolar disorder is a severe, persistent, and recurrent mental illness. Furthermore, an extensive body of literature underscores the pivotal role of sleep disruptions in bipolar disorder [1]. Sleep disturbances are prevalent in both depressive and manic states, and they may manifest even during euthymic periods [1–3]. Sleep disruptions in individuals with bipolar disorder correlate with residual mood symptoms, recurrence of mood episodes, and diminished quality of life [4–8]; therefore, it is imperative to promptly identify the factors associated with sleep disturbance in such individuals.

Light plays a crucial role in nocturnal sleep regulation. Previous studies involving both healthy adults and those with bipolar disorder have indicated that evening or nighttime light exposure negatively affects sleep quality [9–12]. Conversely, daytime light exposure exerts the opposite effect. Various field studies have reported that more daytime light is associated with an earlier sleep–wake phase, shorter sleep latency, higher sleep efficiency, and enhanced sleep quality [13–16]. Furthermore, greater daytime melanopic equivalent daylight illuminance is positively correlated with improved sleep quality at night [17]. This evidence suggests that increased light exposure during daytime may lead to improved sleep. However, to our knowledge, no previous study has investigated whether the level of daytime light exposure in real-life settings is associated with sleep quality in patients with bipolar disorder, in whom there is evidence for disrupted light sensitivity [18]. In addition, previous experimental studies have demonstrated that increased light exposure during the daytime may reduce the sleep–wake cycle effects associated with unavoidable nighttime light exposure [19, 20]. This evidence indicates that daytime and nighttime light exposure interact with each other on the sleep–wake cycle. Given that light exposure in daily life is associated with several symptoms such as altered mood, circadian rhythm, and obesity in bipolar disorder [21–24], the interaction of daytime and nighttime light exposure could affect sleep in those with bipolar disorder.

This cross-sectional study primarily aimed to assess the association between daytime light exposure and sleep (as measured by actigraphy) in a cohort of 204 individuals diagnosed with bipolar disorder. The secondary objective was to examine the effect of the interaction term of daytime and nighttime illuminance on sleep.

## Materials and methods

### Study participants

Between August 2017 and May 2021, we enrolled 218 individuals diagnosed with bipolar disorder aged 18–75 years in the “Association between Pathology of Bipolar Disorder and Light Exposure in Daily Life (APPLE)” cohort study [9]. The Diagnostic and Statistical Manual of Mental Disorders 5th edition criteria were used to diagnose bipolar disorder I or II. The study excluded participants who were nightshift workers, those at high risk of suicide as determined by a trained psychiatrist, and individuals experiencing acute mood episodes.

Demographic and clinical characteristics were assessed at the clinic, and participants were then instructed to adhere to the following guidelines for seven consecutive days: (1) wear an actigraph capable of measuring ambient light (Actiwatch Spectrum Plus; Respironics, Murrysville, PA, USA) on the non-dominant wrist; (2) avoid covering the actigraph with clothing— participants were given an armband to prevent their shirtsleeves from covering the device; (3) keep in a sleep diary; and (4) record bedroom illuminance from bedtime to rise time using a portable light meter (LX-1128SD; Sato Shoji, Kanagawa, Japan). The light sensor in the Actiwatch Spectrum consists of light sensitive photodiodes that measure illuminance from 400–700 nanometers in units of lux and the range is 0.1–35,000 lux. The optical sensor of LX-1128SD has a spectral sensitivity approximating that of the human eye and provides an illuminance sensitivity 0–100,000 lux with ±4% accuracy.

Of the 218 participants, 204 completed actigraphy and bedroom light measurements, maintained a sleep diary for seven consecutive days and underwent clinical assessments.

The authors assert that all procedures contributing to this work comply with the ethical standards of the relevant national and institutional committees on human experimentation and with the Helsinki Declaration of 1975, as revised in 2008. All procedures involving human patients were approved by the Ethics Committee of Okehazama Hospital (identifier: H29-011). Written informed consent was obtained from all patients before participating in the study. The study is registered at the University hospital Medical Information Network Clinical Trials Registry (UMIN-CTR identifier: UMIN000028239).

### Sleep assessment

Sleep was evaluated by actigraphy for seven days. Movement data were collected at 1-min intervals and sleep state (i.e., sleep or wake) was imputed using a standard algorithm [25] with a “moderate” (40 counts/min) sensitivity threshold (Actiware version 6.0.9; Respironics Inc., PA, USA), as suggested for use in those with bipolar disorder [26]. This method of actigraphy analysis has been utilized in prior research involving individuals with bipolar disorder [9, 27]. Time in bed was set by the bedtime and rise time recorded in the sleep diary. The following four sleep parameters were estimated: (1) sleep efficiency—the percentage of total sleep time between bedtime and rise time; (2) sleep onset latency—the time between bedtime and sleep onset; (3) wake after sleep onset—the time spent awake between sleep onset and rise time; and (4) total sleep time—the sleep time between the sleep onset and sleep offset excluding wake after sleep onset.

### Light exposure assessment

Daytime light exposure, from the interval between the participant’s rise time and bedtime, was assessed using an actigraph at 1-min intervals. The actigraph software detection algorithm (Actiware, version 6.0.9, Respironics) automatically excluded intervals when the device was not in contact with the participant’s skin (≥2 mm away). If any of these periods exceeded 50% of the daytime period, the data for that day were excluded from the analysis. Illuminance values below 1 lux during the day were considered artifacts due to clothing coverage and were consequently removed from the analysis [28]. Daytime illuminance was defined as the mean illuminance between rise time and bedtime.

Nighttime light exposure in each participant’s bedroom was assessed using a portable light meter at 1-min intervals from the participant’s bedtime and rise time. Nighttime illuminance was defined as the mean illuminance between bedtime and rise time.

### Covariates

The participants’ depressive and manic states were evaluated using the Montgomery–Åsberg Depression Rating Scale (MADRS) and the Young Mania Rating Scale (YMRS), respectively [29, 30]. Cutoff points for both scales (MADRS: 8 points; YMRS: 8 points) were defined to indicate depressive and manic states per the recommendations of the International Society for Bipolar Disorders Task Force [31]. The first measurement day for each participant, reflecting the duration of daylight from sunrise to sunset in Aichi, Japan (latitude, 35°N), was obtained from the National Astronomical Observatory of Japan’s website. Data concerning participants’ medication regimens, encompassing lithium, anticonvulsants (lamotrigine, valproate, and carbamazepine), sedative antipsychotics (olanzapine, quetiapine, and risperidone), sedative antidepressants (trazodone and mirtazapine), and hypnotics, was retrieved from medical records.

### Statistical analysis

Statistical analyses were conducted using SPSS version 25.0 for Windows (IBM, Armonk, NY, USA). Continuous variables were represented as medians and interquartile ranges, while categorical variables were presented as numbers and percentages. Mean values of seven consecutive days were utilized for analyzing light exposure and sleep parameters. Two-sided P-values < 0.05 were regarded as statistically significant.

Linear regression models incorporated sleep parameters as dependent variables and daytime illuminance parameters as independent variables. In multivariable analysis, simultaneous adjustments were made for sleep associated variables, including age (per year), gender (female/male), current smoking status (yes/no), depressive and manic status (yes/no), day length (per minute), nighttime illuminance (high/low, categorized based on the median value), and the use of sedative medications (sedative atypical antipsychotic, sedative antidepressant, and hypnotics; yes/no). For linear regression models, daytime illuminance and sleep onset latency were analyzed using natural log-transformed continuous variables due to the skewed nature of data distributions. Nighttime illuminance, even after log transformation, remained nonnormally distributed; therefore, a categorical variable based on the median value was used (≥2.28/ <2.28 lux). Interaction of daytime illuminance (x_1_: per log lux) and nighttime illuminance (x_2_: low, 0; high, 1) on sleep parameters (y) were assessed by introducing an interaction term (x_1_ × x_2_) into the linear regression formula: y = β_0_ + β_1_(x_1_) + β_2_(x_2_) + β_3_(x_1_×x_2_) + ε, where β_0_ = intercept, β_1_ = regression coefficient for daytime illuminance, β_2_ = regression coefficient for nighttime illuminance, β_3_ = regression coefficient for the interaction of daytime and nighttime illuminance, and ε = error. In the interaction analysis, daytime illuminance was analyzed after centering to avoid multicollinearity. Multicollinearity was assessed using variance inflation factor.

## Results

The median age of the participants was 44 years, with an interquartile range of 34–53 years, and there were 112 (54.9%) female participants. The median daytime illuminance was 221.8 lux (150.9–306.9 lux), while the nighttime illuminance was 2.3 lux (0.3–9.6 lux) (Table 1). The sleep parameter medians were as follows (Table 1): sleep efficiency, 83.5% (77.1–87.6%); sleep onset latency, 16.0 min (9.1–27.1 min); wake after sleep onset, 55.1 min (39.1–80.3 min); and total sleep time, 386.5 min (334.3–436.9 min).

**Table 1:** Baseline characteristics of the 204 participants.

Characteristics	All (*n* = 204)
Demographic characteristics	
Age, years, median (IQR)	44 (34–53)
Gender, female, n	112 (54.9%)
Married, n	105 (51.5%)
Education (≥13 years), n	120 (58.8%)
Employed, n	82 (40.4%)
Current smoker, n	59 (28.9%)
Clinical characteristics	
Type of bipolar disorder: bipolar disorder I, n	73 (35.8%)
Age at onset of bipolar disorder, years, median (IQR)	31 (22–39)
Duration of illness, years, median (IQR)	11 (7–16)
MADRS score, points, median (IQR)	8 (3.5–14)
YMRS score, points, median (IQR)	2 (0–5)
Bedtime, clock time, median (IQR)	23:11: (22:12–0:15)
Rising time, clock time, and median (IQR)	7:02: (6:14–7:48)
Daytime illuminance, lux, median (IQR)	221.8 (150.9–306.9)
Nighttime illuminance, lux, median (IQR)	2.3 (0.3–9.6)
Medications	
Lithium, n	81 (39.7%)
Anticonvulsant, n	114 (55.9%)
Sedative atypical antipsychotic, n	58 (28.4%)
Sedative antidepressant, n	41 (20.1%)
Hypnotics, n	128 (62.7%)
Sleep parameters	
Sleep efficiency, %, median (IQR)	83.5 (77.1–87.6)
Sleep onset latency, min, median (IQR)	16.0 (9.1–27.1)
Wake after sleep onset, min, median (IQR)	55.1 (39.1–80.3)
Total sleep time, min, median (IQR)	386.5 (334.3–436.9)

Data are expressed as the median (interquartile range) or number (%).

*MADRS* montgomery–åsberg depression rating scale, *YMRS* young mania rating scale.

Univariable linear regression analyses demonstrated the presence of significant associations among increased daytime illuminance, higher sleep efficiency, and shorter sleep onset latency (Crude model: Table 2). In multivariable models, after adjusting for potential confounders, including age, gender, current smoking status, depressive and manic status, day length, nighttime illuminance, and the use of sedative atypical antipsychotics, sedative antidepressants, and hypnotics, greater daytime illuminance was significantly associated with higher sleep efficiency, shorter sleep onset latency, and shorter wake after sleep onset (Adjusted model: Table 2).

**Table 2:** Linear regression results for sleep parameters associated with daytime illuminance (per log lux).

	Crude model				Adjusted model			
Sleep parameters	β	95% CI		*P*	β	95% CI		*P*
Sleep efficiency, %	2.33	0.21	4.44	0.031	3.54	1.43	5.65	0.001
Sleep onset latency, log min	−0.24	−0.43	−0.05	0.013	−0.30	−0.50	−0.10	0.003
Wake after sleep onset, min	−7.14	−16.45	2.16	0.132	−11.00	−20.47	−1.52	0.023
Total sleep time, min	10.68	−12.14	33.50	0.357	17.77	−5.36	40.90	0.131

The daytime illuminance was analyzed after natural logarithmic transformation. Adjusted for age, gender, current smoking status, depressive and manic status, day length, nighttime illuminance, and use of sedative atypical antipsychotics, sedative antidepressants, and hypnotics.

*CI* confidence interval.

**Fig. 1:**
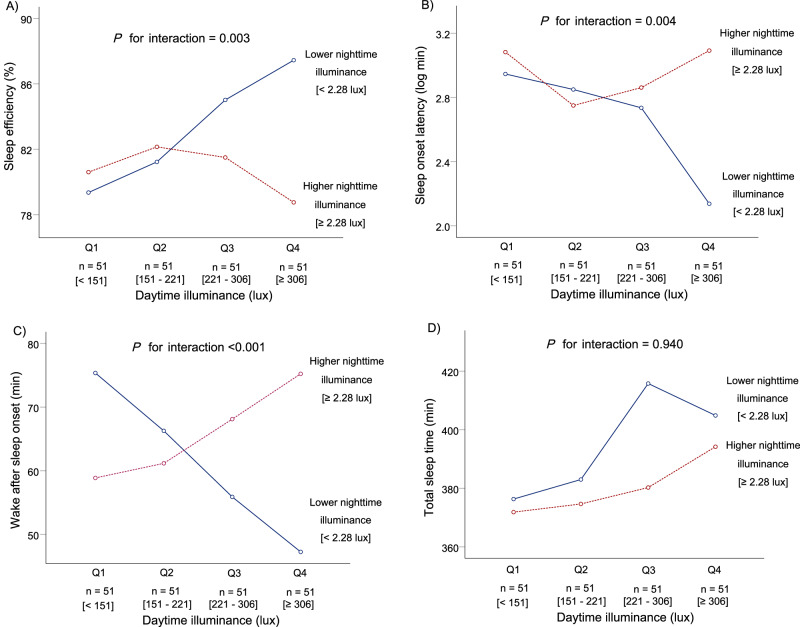
Associations between quartile daytime illuminance and sleep parameters stratified by median nighttime illuminance. Blue solid lines present mean values in each sleep parameter, including sleep efficiency **A**), sleep onset latency **B**), wake after sleep onset **C**), and total sleep time **D**), in the lower nighttime illuminance group. Red dotted lines represent mean values in each sleep parameter in the higher nighttime illuminance group. *P* for interaction presents the interaction of daytime illuminance (per log lux) and nighttime illuminance (high/low) on sleep parameters.

In the subsequent analysis, we assessed the interaction of daytime and nighttime illuminance on sleep parameters. There was no significant multicollinearity detected in any of the multivariable models, as all variance inflation factors were below 10. The graphic representation in Fig. 1 illustrates the associations between quartile daytime illuminance and sleep parameters, stratified by median nighttime illuminance, facilitating the interpretation of statistical results. In the lower nighttime illuminance group (Nighttime illuminance < 2.28 lux), sleep efficiency was higher as increments in daytime illuminance (Fig. 1A), whereas sleep onset latency and wake after sleep onset were shorter (Fig. 1B, C). Conversely, in the higher nighttime illuminance group (Nighttime illuminance ≥ 2.28 lux), sleep efficiency and sleep onset latency were not significantly associated with increasing daytime illuminance (Fig. 1A, B), and wake after sleep onset was longer (Fig. 1C). Total sleep time was longer with higher daytime illuminance in both groups (Fig. 1D). The interaction term of daytime and nighttime illuminance was significantly associated with sleep efficiency, sleep onset latency, and wake after sleep onset (Fig. 1 and Supplementary Table 1).

Table 3 presents the linear regression analysis results for sleep parameters associated with daytime illuminance, stratified by median nighttime illuminance. Daytime illuminance in the lower nighttime illuminance group was significantly associated with higher sleep efficiency, shorter sleep onset latency, and shorter wake after sleep onset, whereas daytime illuminance in the higher nighttime illuminance group was not significantly associated with any sleep parameter.

**Table 3:** Linear regression results for sleep parameters associated with daytime illuminance stratified by nighttime illuminance.

	Nighttime illuminance Low (<2.28 lux)				Nighttime illuminance High (≥2.28 lux)			
Sleep parameters	β	95% CI		*P*	β	95% CI		*P*
Sleep efficiency, %	5.54	2.98	8.10	<0.001	−0.77	−4.09	2.55	0.647
Sleep onset latency, log min	−0.52	−0.78	−0.26	<0.001	0.02	−0.24	0.28	0.874
Wake after sleep onset, min	−22.63	−35.12	−10.14	<0.001	9.11	−4.39	22.61	0.184
Total sleep time, min	11.12	−23.06	45.29	0.520	12.87	−18.16	43.89	0.413

Daytime illuminance was analyzed after natural logarithmic transformation. Nighttime illuminance, even after log transformation, remained nonnormally distributed; therefore, a categorical variable based on the median value was used (≥2.28/<2.28 lux).

*CI* confidence interval.

## Discussion

We demonstrated the existence of a statistically significant association between daytime light exposure and sleep in patients with bipolar disorder. This association remained robust even after adjusting for major potential confounders, including age, gender, smoking status, mood symptoms, day length, nighttime light exposure, and sedative use. Moreover, we identified a significant interaction of both daytime and nighttime light exposure on sleep.

Our analysis aligns with the findings of prior studies that have reported a correlation between daytime light exposure and sleep in individuals not specifically selected for bipolar disorder. For example, a recent field study involving 400,000 participants from the UK Biobank revealed that increased daytime light was associated with a decrease in insomnia symptoms (Odds ratio: 0.94–0.97) [14]. Furthermore, a crossover field study conducted on twelve healthy students indicated that morning bright light exposure, compared with regular office light, led to higher sleep efficiency, reduced fragmentation index, and shorter sleep latency [13]. The findings of our study not only support the outcomes of these earlier investigations but also demonstrate the independence of these results from several potential confounders, including the influence of sedative psychiatric medications. Given that individuals with bipolar disorder have been reported to be hypersensitive to nocturnal melatonin suppression by light, the influence of daytime light exposure on sleep could be stronger in those with bipolar disorder as compared to the healthy people. Further studies to confirm the relationship between daytime light and sleep by comparing those with bipolar disorder and unaffected controls are required.

The impact of bright light therapy on sleep in patients with bipolar disorder remains a subject of debate. In a previous randomized controlled trial investigating bright light therapy in bipolar disorder, Pittsburgh Sleep Quality Index scores significantly improved in the bright light therapy group compared with the placebo group [32]. Conversely, another randomized controlled trial in bipolar disorder reported that despite significant improvement in depressive symptoms in the bright light therapy group compared with the placebo group, the enhancement in sleep quality did not differ significantly [33]. We speculate that the conflicting results in previous studies may be attributed to nighttime light exposure. Our results indicate that the impact of daytime light exposure on sleep varies depending on the level of nighttime light exposure (Table 3). The aforementioned previous studies did not measure nighttime light exposure because they primarily focused on bright light exposure during the daytime. Therefore, in order to reap the improved sleep associated with increased daytime light exposure, avoiding nighttime light exposure be necessary in those with bipolar disorder.

Previous studies have presented evidence that the adverse effects of evening/nighttime light can be alleviated through early-day bright light exposure [19, 20, 34]. Although the contrasting impacts of light exposure during the daytime and evening/nighttime on sleep and circadian function are well established [35, 36], these modulatory effects also extend to more immediate actions of evening light, such as its ability to suppress melatonin. Therefore, our investigation focused on assessing the interaction between daytime and nighttime light exposure concerning sleep in patients with bipolar disorder. As anticipated, the interaction of daytime and nighttime light exposure was statistically significant. Although we did not observe a reduction in the negative effects on sleep associated with nighttime light exposure with increased daytime light exposure, we identified a positive association on sleep quality through the interaction of heightened daytime light and diminished nighttime light.

Although the mechanisms underlying the association between daytime light exposure and sleep in patients with bipolar disorder remain elusive, previous studies have proposed several potential ones. Light exposure is widely acknowledged as the primary synchronizing stimulus for the human circadian system [35, 36]. Daytime light exposure induces circadian-phase advance and increased melatonin secretion [23, 35, 37], which are —both linked to sleep improvement [38, 39]. Another conceivable mechanism is that daytime light exposure may enhance sleep by increasing the amplitude of the circadian rhythm during awakening [40]. A larger circadian activity rhythm could produce both a stronger signal for alertness during the day and a stronger signal for nocturnal sleep [41, 42]. Conversely, nighttime light exposure diminishes amplitude, leading to sleep disturbance. Previous studies have specifically noted that individuals with bipolar disorder show hypersensitivity to nocturnal melatonin secretion induced by light compared with healthy adults [18]. Therefore, sleep disturbance, especially in bipolar disorder, may be attributed to light/dark cycle attenuation.

Our findings indicate that appropriate light exposure could contribute significantly to sleep management in patients with bipolar disorder. Currently, individuals spend most of their time indoors (on average 93%), and recent studies have revealed that people are often exposed to less light during the daytime and more light prebedtime than recommended [43, 44]. Daytime indoor light intensities are typically much lower than outdoor light exposures, whereas evening/nighttime indoor light intensities (from artificial light sources and light-emitting screens) are high relative to outdoor light at night, potentially leading to sleep disturbances. Since many individuals with bipolar disorder are reported to spend much time in a sedentary state [45], they are likely to spend much time indoor more frequently than healthy individuals, resulting in exposure to inappropriate light. Moreover, in bipolar disorder, inappropriate light exposure is not only linked to sleep issues but also contributes to circadian disruption, mood alterations, and metabolic changes [23, 24, 46, 47]. Therefore, appropriate light exposure is paramount in the management of individuals with bipolar disorder.

The strengths of this study include its objective measurement of both light exposure and sleep, coupled with the assessment of the interaction between daytime and nighttime light exposure on sleep. Nevertheless, the study also has some limitations. First, the cross-sectional design of the study prevents us from establishing causality in the association between daytime light exposure and sleep in patients with bipolar disorder. It may also be that the increase in nighttime light exposure was not a cause of the disrupted sleep but rather a consequence. That is, when a participant’s sleep was disrupted, they would turn on a light. Second, the nonrandom selection of study participants may have introduced selection bias, which could impact the robustness of our results. Third, our evaluation of sleep relied on actigraphy. Although various methods (such as sleep diaries, questionnaires, and polysomnography) exist for assessing sleep, polysomnography is considered the gold standard. However, actigraphy was found to be comparable to polysomnography in measuring sleep parameters for patients with bipolar disorder [26]. Additionally, conducting polysomnography in a home setting is challenging. Therefore, the use of actigraphy for sleep evaluation in patients with bipolar disorder is justified. Finally, we measured daytime light exposure over only a seven-day period, which might be considered relatively short. However, we speculate that this timeframe provides an approximate representation of an individual’s behavioral patterns because it encompasses both weekdays and weekends.

Our investigation revealed a significant and positive correlation between daytime light exposure and sleep in patients with bipolar disorder. This association remained statistically significant even after adjusting for potential confounders. Notably, we observed a potential interaction between daytime and nighttime light exposure on sleep.

## Supplementary information

Supplemental Table 1. Interaction effect of daytime and nighttime light exposure on sleep parameters

## Data Availability

The data supporting the findings of this study are available from the corresponding author upon reasonable request.

## References

[1] Harvey AG. Sleep and circadian rhythms in bipolar disorder: seeking synchrony, harmony, and regulation. Am J Psychiatry. 2008;165:820–9. 18519522 10.1176/appi.ajp.2008.08010098

[2] Geoffroy PA, Scott J, Boudebesse C, Lajnef M, Henry C, Leboyer M, et al. Sleep in patients with remitted bipolar disorders: a meta-analysis of actigraphy studies. Acta Psychiatr Scand. 2015;131:89–99. 25430914 10.1111/acps.12367

[3] Ng TH, Chung KF, Ho FY, Yeung WF, Yung KP, Lam TH. Sleep-wake disturbance in interepisode bipolar disorder and high-risk individuals: a systematic review and meta-analysis. Sleep Med Rev. 2015;20:46–58. 25060968 10.1016/j.smrv.2014.06.006

[4] Cretu JB, Culver JL, Goffin KC, Shah S, Ketter TA. Sleep, residual mood symptoms, and time to relapse in recovered patients with bipolar disorder. J Affect Disord. 2016;190:162–6. 26519636 10.1016/j.jad.2015.09.076

[5] Gershon A, Do D, Satyanarayana S, Shah S, Yuen LD, Hooshmand F, et al. Abnormal sleep duration associated with hastened depressive recurrence in bipolar disorder. J Affect Disord. 2017;218:374–9. 28500982 10.1016/j.jad.2017.05.015 PMC6389505

[6] Sylvia LG, Dupuy JM, Ostacher MJ, Cowperthwait CM, Hay AC, Sachs GS, et al. Sleep disturbance in euthymic bipolar patients. J Psychopharmacol. 2012;26:1108–12. 21965189 10.1177/0269881111421973 PMC3787715

[7] Eidelman P, Talbot LS, Gruber J, Harvey AG. Sleep, illness course, and concurrent symptoms in inter-episode bipolar disorder. J Behav Ther Exp Psychiatry. 2010;41:145–9. 20004888 10.1016/j.jbtep.2009.11.007 PMC2824048

[8] Gruber J, Harvey AG, Wang PW, Brooks JO 3rd, Thase ME, et al. Sleep functioning in relation to mood, function, and quality of life at entry to the systematic treatment enhancement program for bipolar disorder (STEP-BD). J Affect Disord. 2009;114:41–49. 18707765 10.1016/j.jad.2008.06.028 PMC2677624

[9] Esaki Y, Kitajima T, Obayashi K, Saeki K, Fujita K, Iwata N. Light exposure at night and sleep quality in bipolar disorder: The APPLE cohort study. J Affect Disord. 2019;257:314–20. 31302520 10.1016/j.jad.2019.07.031

[10] Esaki Y, Obayashi K, Saeki K, Fujita K, Iwata N, Kitajima T. Effect of evening light exposure on sleep in bipolar disorder: a longitudinal analysis for repeated measures in the APPLE cohort. Aust N Z J Psychiatry. 2021;55:305–13. 33118369 10.1177/0004867420968886

[11] Obayashi K, Saeki K, Iwamoto J, Okamoto N, Tomioka K, Nezu S, et al. Effect of exposure to evening light on sleep initiation in the elderly: a longitudinal analysis for repeated measurements in home settings. Chronobiol Int. 2014;31:461–7. 24147658 10.3109/07420528.2013.840647

[12] Obayashi K, Saeki K, Kurumatani N. Association between light exposure at night and insomnia in the general elderly population: the HEIJO-KYO cohort. Chronobiol Int. 2014;31:976–82. 25025617 10.3109/07420528.2014.937491

[13] He M, Ru T, Li S, Li Y, Zhou G. Shine light on sleep: morning bright light improves nocturnal sleep and next morning alertness among college students. J Sleep Res. 2023;32:e13724. 36058557 10.1111/jsr.13724

[14] Burns AC, Saxena R, Vetter C, Phillips AJK, Lane JM, Cain SW. Time spent in outdoor light is associated with mood, sleep, and circadian rhythm-related outcomes: a cross-sectional and longitudinal study in over 400,000 UK Biobank participants. J Affect Disord. 2021;295:347–52. 34488088 10.1016/j.jad.2021.08.056 PMC8892387

[15] Figueiro MG, Steverson B, Heerwagen J, Kampschroer K, Hunter CM, Gonzales K, et al. The impact of daytime light exposures on sleep and mood in office workers. Sleep Health. 2017;3:204–15. 28526259 10.1016/j.sleh.2017.03.005

[16] Nagare R, Woo M, MacNaughton P, Plitnick B, Tinianov B, Figueiro M. Access to daylight at home improves circadian alignment, sleep, and mental health in healthy adults: a crossover study. Int J Environ Res Public Health. 2021;18:9980. 34639284 10.3390/ijerph18199980 PMC8507741

[17] Brown TM, Brainard GC, Cajochen C, Czeisler CA, Hanifin JP, Lockley SW, et al. Recommendations for daytime, evening, and nighttime indoor light exposure to best support physiology, sleep, and wakefulness in healthy adults. PLoS Biol. 2022;20:e3001571. 35298459 10.1371/journal.pbio.3001571 PMC8929548

[18] Roguski A, Ritter P, Smith DJ. Sensitivity to light in bipolar disorder: implications for research and clinical practice. Br J Psychiatry. 2024;224:143–6. 38174418 10.1192/bjp.2023.150 PMC7615859

[19] Smith KA, Schoen MW, Czeisler CA. Adaptation of human pineal melatonin suppression by recent photic history. J Clin Endocrinol Metab. 2004;89:3610–4. 15240654 10.1210/jc.2003-032100

[20] Hebert M, Martin SK, Lee C, Eastman CI. The effects of prior light history on the suppression of melatonin by light in humans. J Pineal Res. 2002;33:198–203. 12390501 10.1034/j.1600-079x.2002.01885.x PMC3925650

[21] Esaki Y, Obayashi K, Saeki K, Fujita K, Iwata N, Kitajima T. Preventive effect of morning light exposure on relapse into depressive episode in bipolar disorder. Acta Psychiatr Scand. 2021;143:328–38. 33587769 10.1111/acps.13287

[22] Esaki Y, Obayashi K, Saeki K, Fujita K, Iwata N, Kitajima T. Effect of nighttime bedroom light exposure on mood episode relapses in bipolar disorder. Acta Psychiatr Scand. 2022;146:64–73. 35253206 10.1111/acps.13422

[23] Esaki Y, Obayashi K, Saeki K, Fujita K, Iwata N, Kitajima T. Habitual light exposure and circadian activity rhythms in bipolar disorder: a cross-sectional analysis of the APPLE cohort. J Affect Disord. 2023;323:762–9. 36538951 10.1016/j.jad.2022.12.034

[24] Esaki Y, Obayashi K, Saeki K, Fujita K, Iwata N, Kitajima T. Bedroom light exposure at night and obesity in individuals with bipolar disorder: a cross-sectional analysis of the APPLE cohort. Physiol Behav. 2021;230:113281. 33306979 10.1016/j.physbeh.2020.113281

[25] Cole RJ, Kripke DF, Gruen W, Mullaney DJ, Gillin JC. Automatic sleep/wake identification from wrist activity. Sleep. 1992;15:461–9. 1455130 10.1093/sleep/15.5.461

[26] Kaplan KA, Talbot LS, Gruber J, Harvey AG. Evaluating sleep in bipolar disorder: comparison between actigraphy, polysomnography, and sleep diary. Bipolar Disord. 2012;14:870–9. 23167935 10.1111/bdi.12021 PMC3549461

[27] Boudebesse C, Leboyer M, Begley A, Wood A, Miewald J, Hall M, et al. Comparison of five actigraphy scoring methods with bipolar disorder. Behav Sleep Med. 2013;11:275–82. 23205606 10.1080/15402002.2012.685997 PMC3869094

[28] Scheuermaier K, Laffan AM, Duffy JF. Light exposure patterns in healthy older and young adults. J Biol Rhythms. 2010;25:113–22. 20348462 10.1177/0748730410361916 PMC2847788

[29] Montgomery SA, Asberg M. A new depression scale designed to be sensitive to change. Br J Psychiatry. 1979;134:382–9. 444788 10.1192/bjp.134.4.382

[30] Young RC, Biggs JT, Ziegler VE, Meyer DA. A rating scale for mania: reliability, validity and sensitivity. Br J Psychiatry. 1978;133:429–35. 728692 10.1192/bjp.133.5.429

[31] Tohen M, Frank E, Bowden CL, Colom F, Ghaemi SN, Yatham LN, et al. The International Society for Bipolar Disorders (ISBD) Task Force report on the nomenclature of course and outcome in bipolar disorders. Bipolar Disord. 2009;11:453–73. 19624385 10.1111/j.1399-5618.2009.00726.x

[32] Yorguner Kupeli N, Bulut NS, Carkaxhiu Bulut G, Kurt E, Kora K. Efficacy of bright light therapy in bipolar depression. Psychiatry Res. 2018;260:432–8. 29268206 10.1016/j.psychres.2017.12.020

[33] Sit DK, McGowan J, Wiltrout C, Diler RS, Dills JJ, Luther J, et al. Adjunctive bright light therapy for bipolar depression: a randomized double-blind placebo-controlled trial. Am J Psychiatry. 2018;175:131–9. 28969438 10.1176/appi.ajp.2017.16101200

[34] Te Kulve M, Schlangen LJM, van Marken Lichtenbelt WD. Early evening light mitigates sleep compromising physiological and alerting responses to subsequent late evening light. Sci Rep. 2019;9:16064. 31690740 10.1038/s41598-019-52352-w PMC6831674

[35] Khalsa SB, Jewett ME, Cajochen C, Czeisler CA. A phase response curve to single bright light pulses in human subjects. J Physiol. 2003;549:945–52. 12717008 10.1113/jphysiol.2003.040477 PMC2342968

[36] St Hilaire MA, Gooley JJ, Khalsa SB, Kronauer RE, Czeisler CA, Lockley SW. Human phase response curve to a 1 h pulse of bright white light. J Physiol. 2012;590:3035–45. 22547633 10.1113/jphysiol.2012.227892 PMC3406389

[37] Obayashi K, Saeki K, Iwamoto J, Okamoto N, Tomioka K, Nezu S, et al. Positive effect of daylight exposure on nocturnal urinary melatonin excretion in the elderly: a cross-sectional analysis of the HEIJO-KYO study. J Clin Endocrinol Metab. 2012;97:4166–73. 22948764 10.1210/jc.2012-1873

[38] Duffy JF, Wang W, Ronda JM, Czeisler CA. High dose melatonin increases sleep duration during nighttime and daytime sleep episodes in older adults. J Pineal Res. 2022;73:e12801. 35436355 10.1111/jpi.12801 PMC9288519

[39] Xie Z, Chen F, Li WA, Geng X, Li C, Meng X, et al. A review of sleep disorders and melatonin. Neurol Res. 2017;39:559–65. 28460563 10.1080/01616412.2017.1315864

[40] Jewett ME, Kronauer RE, Czeisler CA. Phase-amplitude resetting of the human circadian pacemaker via bright light: a further analysis. J Biol Rhythms. 1994;9:295–314. 7772797 10.1177/074873049400900310

[41] Ancoli-Israel S, Gehrman P, Martin JL, Shochat T, Marler M, Corey-Bloom J, et al. Increased light exposure consolidates sleep and strengthens circadian rhythms in severe Alzheimer’s disease patients. Behav Sleep Med. 2003;1:22–36. 15600135 10.1207/S15402010BSM0101_4

[42] Gonzalez MM, Aston-Jones G. Circadian regulation of arousal: role of the noradrenergic locus coeruleus system and light exposure. Sleep. 2006;29:1327–36. 17068987 10.1093/sleep/29.10.1327

[43] Klepeis NE, Nelson WC, Ott WR, Robinson JP, Tsang AM, Switzer P, et al. The National Human Activity Pattern Survey (NHAPS): a resource for assessing exposure to environmental pollutants. J Expo Anal Environ Epidemiol. 2001;11:231–52. 11477521 10.1038/sj.jea.7500165

[44] Didikoglu A, Mohammadian N, Johnson S, van Tongeren M, Wright P, Casson AJ, et al. Associations between light exposure and sleep timing and sleepiness while awake in a sample of UK adults in everyday life. Proc Natl Acad Sci USA. 2023;120:e2301608120. 37812713 10.1073/pnas.2301608120 PMC10589638

[45] Vancampfort D, Firth J, Schuch FB, Rosenbaum S, Mugisha J, Hallgren M, et al. Sedentary behavior and physical activity levels in people with schizophrenia, bipolar disorder and major depressive disorder: a global systematic review and meta-analysis. World Psychiatry. 2017;16:308–15. 28941119 10.1002/wps.20458 PMC5608847

[46] Esaki Y, Kitajima T, Obayashi K, Saeki K, Fujita K, Iwata N. Daytime light exposure in daily life and depressive symptoms in bipolar disorder: a cross-sectional analysis in the APPLE cohort. J Psychiatr Res. 2019;116:151–6. 31247358 10.1016/j.jpsychires.2019.06.010

[47] Esaki Y, Obayashi K, Saeki K, Fujita K, Iwata N, Kitajima T. Association between light exposure at night and manic symptoms in bipolar disorder: cross-sectional analysis of the APPLE cohort. Chronobiol Int. 2020;37:887–96. 32238002 10.1080/07420528.2020.1746799

