# Supplementary material for: Interaction of daytime and nighttime light exposure on objective sleep quality in patients with bipolar disorder: a cross-sectional analysis of the APPLE cohort
Source: Transl Psychiatry. 2025 Aug 18;15:291. doi: 10.1038/s41398-025-03549-3 (PMC12361580; doi:10.1038/s41398-025-03549-3)
Supplement: Supplementary file 1 — Supplemental Table 1. Interaction effect of daytime and nighttime light exposure on sleep parameters [file 41398_2025_3549_MOESM1_ESM.docx]

| **Supplemental Table 1.** Interaction effect of daytime and nighttime light exposure on sleep parameters | | | | | |
| --- | --- | --- | --- | --- | --- |
| Dependent variables  (Sleep parameters) | Independent variables | β | 95% CI | | *P* |
| Sleep efficiency, % | Daytime light (per log lux) | 5.54 | 2.69 | 8.39 | <0.001 |
|  | Nighttime light (high/low) | −2.50 | −4.90 | −0.10 | 0.041 |
|  | Interaction (daytime light * nighttime light) | −6.31 | −10.45 | −2.17 | 0.003 |
| Sleep onset latency, log min | Daytime light (per log lux) | −0.52 | −0.77 | −0.27 | <0.001 |
|  | Nighttime light (high/low) | 0.28 | 0.07 | 0.49 | 0.011 |
|  | Interaction (daytime light * nighttime light) | 0.54 | 0.18 | 0.91 | 0.004 |
| Wake after sleep onset, min | Daytime light (per log lux) | −22.63 | −35.2 | −10.05 | <0.001 |
|  | Nighttime light (high/low) | 4.92 | −5.65 | 15.50 | 0.359 |
|  | Interaction (daytime light * nighttime light) | 31.75 | 13.47 | 50.1 | <0.001 |
| Total sleep time, min | Daytime light (per log lux) | 11.12 | −20.58 | 42.81 | 0.940 |
|  | Nighttime light (high/low) | −14.10 | −40.77 | 12.56 | 0.298 |
|  | Interaction (daytime light * nighttime light) | 1.75 | −44.30 | 47.80 | 0.940 |
| Daytime illuminance was analyzed after natural logarithmic transformation and centering. CI, confidence interval | | | | | |
